# Sylvian Arteriovenous Malformation Resection and Associated Middle Cerebral Artery Aneurysm Clipping: Technical Nuances of Concurrent Surgical Treatment

**DOI:** 10.7759/cureus.2166

**Published:** 2018-02-07

**Authors:** Dale Ding, Thomas J Buell, Daniel M Raper, Ching-Jen Chen, Panagiotis Mastorakos, Kenneth C Liu, Dennis G Vollmer

**Affiliations:** 1 Neurosurgery, Barrow Neurological Institute, St. Joseph's Hospital and Medical Center, Phoenix, AZ, USA; 2 Department of Neurological Surgery, University of Virginia; 3 Department of Neurological Sugery, University of Virginia, Charlottesville, United States; 4 Department of Neurological Surgery, University of Virginia; 5 Department of Neurological Surgery, University of Virginia, Charlottesville, United States

**Keywords:** intracranial aneurysm, intracranial arteriovenous malformation, microsurgery, middle cerebral artery, sylvian fissure, vascular malformations

## Abstract

Approximately 10-30% of brain arteriovenous malformations (AVM) have associated arterial aneurysms (AAA), and the management of these lesions can be challenging. In this video technical note, we demonstrate the microsurgical treatment of an unruptured, Spetzler-Martin grade I AVM in the distal Sylvian fissure with two AAAs arising proximally from the inferior M2 trunk immediately distal to the middle cerebral artery (MCA) bifurcation. First, we resected the superficially located AVM to normalize the blood flow through the Sylvian vein. Next, we performed a Sylvian fissure dissection to access and clip the two MCA AAAs. We also discuss the technical nuances of tandem surgical intervention for AVMs with AAAs as it pertains to this case, particularly with respect to the order of lesion treatment, Sylvian fissure dissection, nidal resection, and aneurysm clipping.

## Introduction

Brain arteriovenous malformations (AVM) harbor associated aneurysms arising from feeding arteries in approximately 10-30% of cases [1, 2]. Redekop et al. classified AVM-associated arterial aneurysms (AAA), according to their location, as intranidal (type I), proximal flow-related (type IIa), distal flow-related (type IIb), and unrelated (type III) [1]. Flow-related, or prenidal, AAAs more frequently arise proximally on the feeding artery than distally, and these proximal locations include the supraclinoid internal carotid artery, circle of Willis, anterior cerebral artery up to the anterior communicating artery, middle cerebral artery (MCA) up to its bifurcation, and vertebrobasilar trunk [1, 2]. Proximal prenidal AAAs may be located distant to the AVM, such that dissection of the nidus does not expose the AAA. Therefore, these uncommon lesions pose unique challenges to the cerebrovascular surgeon. In this technical note, we describe the concurrent surgical treatment of a distal Sylvian AVM with two proximal prenidal AAAs arising in the vicinity of the MCA bifurcation.

## Technical report

The preoperative imaging, critical portions of the procedure, and postoperative imaging are shown and narrated in Video 1.

**Video 1:** Sylvian arteriovenous malformation resection and associated middle cerebral artery aneurysm clipping. Narrative video describing the technical nuances of concurrent surgical treatment of an unruptured, right-sided Spetzler-Martin grade I AVM of the distal Sylvian fissure with two proximal prenidal AVM-associated aneurysms arising from the inferior M2 trunk immediately distal to the MCA bifurcation. AVM: Arteriovenous malformations; MCA: Middle cerebral artery.

A 77-year-old male presented with seizures, and noninvasive neuroimaging showed an unruptured vascular malformation. Preoperative catheter cerebral angiography showed a right-sided, Spetzler-Martin grade I AVM located in the distal Sylvian fissure. The AVM was supplied by distal branches of the MCA, and drained both anteriorly through the Sylvian vein and posteriorly through the vein of Labbe. At the junction of the two draining veins, there was a venous varix. There were also two prenidal AAAs arising proximally from the inferior M2 trunk, immediately distal to the MCA bifurcation, with the larger of two AAAs located closer to the bifurcation. After discussing the management options with the patient, he elected to undergo concurrent surgical treatment of both the Sylvian AVM and MCA AAAs.

The patient was positioned in a standard fashion for a right-sided pterional craniotomy, as previously described [3], except with slightly less neck extension and more contralateral head rotation to the left (approximately 60 degrees), so that the distal Sylvian AVM and proximal prenidal MCA AAAs could be accessed through the same craniotomy. We performed a large, right-sided frontotemporal craniotomy which incorporated the lesser wing of the sphenoid anteriorly. The lesser wing of the sphenoid was flattened with rongeurs and a high-speed drill prior to dural opening, in order to facilitate subsequent dissection of the proximal Sylvian fissure.

Upon dural opening, the AVM’s venous varix was immediately noted at the distal end of the Sylvian fissure. Since the varix was overlying the AVM nidus, we initially performed a subarachnoid dissection of the distal Sylvian fissure around the two superficial draining veins and varix. As we circumferentially dissected the AVM’s superficial venous components, elevation of the varix revealed the underlying nidus. Using standard microsurgical technique, we sequentially dissected, coagulated and sharply divided the arterial feeders while preserving the two primary draining veins. Upon adequate devascularization of the nidus, the anterior vein draining into the Sylvian vein was coagulated and divided, which allowed posterior and inferior mobilization of the nidus. Lastly, the posterior vein draining into the vein of Labbe, which was the final vessel tethering the nidus, was ligated. This allowed completion of the AVM resection.

After extirpation of the AVM, we then turned our attention to the MCA AAAs. Since dissection of the AVM had already released cerebrospinal fluid (CSF) from, and hence deflated, the Sylvian fissure, a subfrontal retractor was placed. This maneuver provided gentle tension on the proximal Sylvian fissure and stretched its superficial arachnoid bands taut, which facilitated subsequent dissection. Once the superficial arachnoid of the Sylvian fissure was opened sharply, the fissure was split in an inside-to-out direction. Dissection of the deep portion of the Sylvian fissure exposed the MCA M1 segment, which was followed distally by the MCA bifurcation and both M2 trunks. Both AAAs arose from the inferior M2 trunk, and their anatomy was exposed using standard microsurgical technique. The larger, more proximal M2 AAA had an atherosclerotic neck, so it was under-clipped with a J-shaped aneurysm clip. Finally, the smaller, more distal M2 AAA was clipped with a straight mini-clip.

Intraoperative indocyanine green (ICG) video angiography showed normal filling of both M2 trunks, as well as occlusion of both AAAs. Postoperative angiogram showed complete obliteration of AVM and occlusion of both MCA AAAs. The patient had an uncomplicated postoperative course, and was discharged home on postoperative day 7 without focal neurological deficits.

## Discussion

In light of the recent findings from A Randomized Trial of Unruptured Brain AVMs and the Scottish Audit of Intracranial Vascular Malformations prospective AVM cohort study [4, 5], the management of unruptured AVMs, particularly with regard to the benefit of intervention, has become highly controversial [4-12]. Although conservative management was considered in our patient, due to his old age and lack of prior AVM hemorrhage, we ultimately decided to offer intervention based on two factors: (1) the presence of an AAA has been shown to increase an AVM’s hemorrhage risk [1, 13, 14], and (2) the patient presented with AVM-associated seizures, which can be ameliorated or abolished upon nidal obliteration [15-20]. Targeting the AVM with stereotactic radiosurgery would have been a reasonable treatment option [2, 6-8, 10, 15-17, 19]. However, due to the wide-necked morphology of the two AAAs, we did not believe they could be embolized without stent assistance, which would have necessitated dual antiplatelet therapy in a patient with a patent AVM. After discussing the risks and benefits of conservative management, nonsurgical intervention, and surgical treatment, the patient opted to proceed with surgery.

We present a unique case of a distal Sylvian AVM with two proximal prenidal AAAs arising from the inferior M2 trunk. In the following, we will seek to delineate the following technical nuances: (1) order of AVM and AAA treatment, (2) Sylvian fissure dissection, (3) AVM resection, and (4) AAA clipping. While AAAs are always related to the feeding arteries of the nidus, in the present case, the AVM’s venous drainage affected the approach to the AAA. That is, the Sylvian vein comprised the anterior component of the nidal venous drainage, and dissection of the Sylvian fissure deep to this vein was necessary to expose the two AAAs. Therefore, while the AAAs could have been treated first, injury to the arterialized Sylvian vein draining the AVM would have resulted in considerable bleeding. Furthermore, inadvertent occlusion of the Sylvian vein during Sylvian fissure dissection, prior to AVM resection, would have increased the intranidal pressure and potentially caused an intraoperative rupture of the AVM before adequate dissection of the nidus and control of its arterial feeders.

Thorough dissection of the Sylvian fissure was crucial to minimizing pial transgression and parenchymal injury during exposure of both the AVM and AAAs. We utilize the “inside-out” technique of Sylvian fissure dissection when feasible, which has been endorsed and described in detail by numerous experienced cerebrovascular surgeons [3]. In brief, upon initial opening of the superficial Sylvian arachnoid, one first follows a distal MCA branch into the Sylvian cistern, and then retracts, thereby applying tension on the remainder of the superficial Sylvian arachnoid from deep within the fissure. From inside the Sylvian cistern, the stretched superficial arachnoid of the distal and proximal portions of the Sylvian fissure appears like a canopy, which can then readily be opened with sharp dissection.

Resection of the AVM in our case was fairly routine, except that one had to be prepared to circumdissect and mobilize the venous varix in order to expose the underlying nidus. In the process of dissecting the arachnoid surrounding the varix, it was critically important to preserve the venous outflow of the nidus until it could be thoroughly devascularized. Additionally, any en passage arteries within the distal Sylvian fissure had to be protected, in order to avoid collateral damage to normal cortex. With respect to the AAAs, we noted significant atherosclerosis within the neck of the larger aneurysm, and therefore decided to under-clip this one. For atherosclerotic aneurysms, attaining what appears externally to be the ideal clip placement can, in fact, internally push atheroma into the parent vessel, causing flow-limiting stenosis, parenchymal ischemia, and thromboembolic complications. Under-clipping, as was employed in our case, preserved normal flow through the parent M2 trunk while completely occluding the AAA, as evidenced by both intraoperative ICG and postoperative catheter angiography.

## Conclusions

Only a minority of AVMs harbor AAAs, and these uncommon cases can present unusual technical challenges to the cerebrovascular surgeon, particularly when the AVM and AAA are anatomically distant, such that both lesions cannot be exposed with the same microsurgical dissection. We critically assess the technical nuances of resecting a distal Sylvian AVM and clipping two proximal prenidal AAAs arising from the MCA inferior M2 trunk, with a focus on the order of lesion treatment, as well as dissection of the Sylvian fissure, AVM, and AAAs. Patient, AVM, and AAA factors must be considered in the management of AVMs with AAAs. For appropriately selected AVMs with AAAs, surgical intervention can concurrently afford nidal obliteration and aneurysm occlusion in the same procedure.

## References

[1] Redekop G, TerBrugge K, Montanera W (1998). Arterial aneurysms associated with cerebral arteriovenous malformations: classification, incidence, and risk of hemorrhage. J Neurosurg.

[2] Ding D, Xu Z, Starke RM (2016). Radiosurgery for cerebral arteriovenous malformations with associated arterial aneurysms. World Neurosurg.

[3] Rodriguez-Hernandez A, Sughrue ME, Akhavan S (2013). Current management of middle cerebral artery aneurysms: surgical results with a "clip first" policy. Neurosurgery.

[4] Al-Shahi Salman R, White PM, Counsell CE (2014). Outcome after conservative management or intervention for unruptured brain arteriovenous malformations. JAMA.

[5] Mohr JP, Parides MK, Stapf C (2014). Medical management with or without interventional therapy for unruptured brain arteriovenous malformations (ARUBA): a multicentre, non-blinded, randomised trial. Lancet.

[6] Ding D, Starke RM, Kano H (2017). Radiosurgery for unruptured brain arteriovenous malformations: an international multicenter retrospective cohort study. Neurosurgery.

[7] Ding D, Starke RM, Kano H (2016). Radiosurgery for cerebral arteriovenous malformations in a randomized trial of unruptured brain arteriovenous malformations (ARUBA)-eligible patients: a multicenter study. Stroke.

[8] Ding D, Starke RM, Kano H (2017). Stereotactic radiosurgery for a randomized trial of unruptured brain arteriovenous malformations (ARUBA)-eligible Spetzler-Martin grade I and II arteriovenous malformations: a multicenter study. World Neurosurg.

[9] Ding D, Xu Z, Yen CP (2015). Radiosurgery for unruptured cerebral arteriovenous malformations in pediatric patients. Acta Neurochir.

[10] Ding D, Yen CP, Xu Z (2013). Radiosurgery for patients with unruptured intracranial arteriovenous malformations. J Neurosurg.

[11] Hong CS, Peterson EC, Ding D (2016). Intervention for A randomized trial of unruptured brain arteriovenous malformations (ARUBA) - eligible patients: an evidence-based review. Clin Neurol Neurosurg.

[12] Starke RM, Sheehan JP, Ding D (2014). Conservative management or intervention for unruptured brain arteriovenous malformations. World Neurosurg.

[13] Brown RD, Jr. Jr., Wiebers DO, Forbes GS (1990). Unruptured intracranial aneurysms and arteriovenous malformations: frequency of intracranial hemorrhage and relationship of lesions. J Neurosurg.

[14] Gross BA, Du R (2013). Natural history of cerebral arteriovenous malformations: a meta-analysis. J Neurosurg.

[15] Chen CJ, Chivukula S, Ding D (2014). Seizure outcomes following radiosurgery for cerebral arteriovenous malformations. Neurosurg Focus.

[16] Ding D, Quigg M, Starke RM (2015). Radiosurgery for temporal lobe arteriovenous malformations: effect of temporal location on seizure outcomes. J Neurosurg.

[17] Ding D, Quigg M, Starke RM (2015). Cerebral arteriovenous malformations and epilepsy, part 2: predictors of seizure outcomes following radiosurgery. World Neurosurg.

[18] Ding D, Starke RM, Quigg M (2015). Cerebral arteriovenous malformations and epilepsy, part 1: predictors of seizure presentation. World Neurosurg.

[19] Przybylowski CJ, Ding D, Starke RM (2015). Seizure and anticonvulsant outcomes following stereotactic radiosurgery for intracranial arteriovenous malformations. J Neurosurg.

[20] Wang JY, Yang W, Ye X (2013). Impact on seizure control of surgical resection or radiosurgery for cerebral arteriovenous malformations. Neurosurgery.

